# Mercurial Cream for Injection in Syphilis

**Published:** 1908-04-18

**Authors:** 


### MERCURIAL CREAM FOR INJECTION IN SYPHILIS.

There is a growing tendency to treat syphilis, particularly severe syphilis, in its earlier stages, by the intramuscular injection of mercury preparations into the buttocks. This method of treatment is, and has been, extensively employed in the Army with great success, and Colonel Lambkin's cream of mercury used for the purpose is well enough known.

The main objection to the treatment is its painfulness; although, in order to get rid of syphilis rapidly and thoroughly, it is well worth while to submit to a certain amount of pain. This pain, however, is necessarily influenced to some extent by the volume of material injected. It is therefore likely that the more concentrated the mercury the less bulky will each dose need to be, and the less will the patient suffer provided that the concentration does not itself have deleterious effects.

We are indebted to the *Pharmaceutical Journal* for a formula for mercurial cream, used in ships that visit different climes, of greater strength than those associated with the names of Colonel F. J. Lambkin and Dr. Julius Althaus, and said to be even more satisfactory in use than are the other preparations. It is as follows:—

| | |
|---|---|
| Mercury ... ... ... ... | 20 parts by weight |
| Wool fat ... ... ... ... | 35 parts by weight |
| Chlorbutol ... ... ... | 2 parts by weight |

Liquid paraffin, sufficient to produce 100 parts by measure.

5 minims of the cream contain 1 grain of mercury.

The mercury is rubbed down with a portion of the wool fat in a warm mortar until no globules are visible with a hand lens; the remainder of the wool fat is then added. The chlorbutol is dissolved in a portion of the liquid paraffin by the aid of gentle heat; mixed with the wool fat and mercury; and made up to 100 parts by measure with more liquid paraffin. The whole is heated on a water-bath until just fluid, then strained through three or four layers of white gauze, and stirred with a glass rod until cold.

It is essential that the wool fat used should have been purified by being heated with about twice its volume of water, stirred well, strained through lint, the mixture being then allowed to cool in an ice-chest. The cake of wool fat separated is then scraped free from any brown deposit on both its surfaces. This purifying operation should be repeated until the fat is perfectly clean.

